# Prevalence and factors associated with gastroesophageal reflux disease among primary health care attendants at Abha city, southwestern Saudi Arabia

**DOI:** 10.1016/j.jsps.2021.04.020

**Published:** 2021-04-24

**Authors:** Mohammed A. Alsaleem, Nabil J. Awadalla, Shehata F. Shehata, Awad Saeed Alsamghan, Mohammed A. AlFlan, Marwan M. Alhumaidi, Mohamed S. Alwadai, Fahad S. Althabet, Mohamad S. Alzahrani, Safar A. Alsaleem, Ahmed A. Mahfouz

**Affiliations:** a Department of Family and Community Medicine, College of Medicine, King Khalid University, Abha 61421, Saudi Arabia; b Department of Community Medicine, College of Medicine, Mansoura University, Mansoura 35516, Egypt; c Medical Intern, College of Medicine, King Khalid University, Abha 61421, Saudi Arabia; d Department of Biostatistics, High Institute of Public Health, Alexandria University, Alexandria 21511, Egypt; e Department of Epidemiology, High Institute of Public Health, Alexandria University, Alexandria 21511, Egypt

**Keywords:** Gastroesophageal reflux disease, Prevalence, Associated factors, Saudi Arabia

## Abstract

**Background:**

Gastroesophageal reflux disease (GERD) is an abnormal reflux of the gastric content into the esophagus. In Saudi Arabia the GERD prevalence is not recently well studied.

**Objective:**

To investigate the prevalence of gastroesophageal reflux disease and associated factors among attendants of primary health care centers (PHCCs) at Abha city, Saudi Arabia.

**Method:**

A descriptive cross- sectional study was performed using GerdQ as diagnostic tool for the GERD. The GerdQ consisted of six questions. Four questions were about the positive GERD predictors. The other two questions were about the negative GERD predictors. The scoring of GerdQ relies on the frequency of GERD symptoms during the last seven days. Using stratified random sample technique a representative sample was slected from the study PHCCs taking into conmsideration the relative catchment population in each center among adult males and females attending the selected PHCCs for any reason.

**Results:**

The present study included 320 persons. The study showed a prevalence of GERD of 67.8%. The prevalence of GERD with high impact on daily life (HIDL) was found to be 50%. By multiple logistic regression (enter method) only four significant independent factors associated with GERD were identified; being unmarried (aOR = 1.85, 95% CI:1.02–3.23); smoking (aOR = 2.11, 95% CI: 1.41–5.98), fast food intake (OR = 1.28, 95% CI:1.01–1.71), and subjective perception of stress (OR = 3.0, 95% CI:1.68–5.26).

**Conclusions:**

GERD is a public health problem among adults in the region. Community level awareness programs are recommended. Healthcare providers must be aware of community perceptions and practices.

## 1 Introduction

Gastroesophageal reflux disease (GERD) is defined as an abnormal reflux of the gastric content into the esophagus. In most patients suffering from GERD, the reflux of gastric juice commonly induces heartburn and regurgitations (Bredenoord et al., 2013).

In Saudi Arabia the GERD prevalence is not well studied. Recent studies in Arar and Riyadh reported a prevalence rate of 29% and 45%, respectively (Almadi et al., 2014, Alsulobi et al., 2017). Prevalence of GERD increases in most western countries and are rising in some developed Asian countries. Many recent studies have clarified that nighttime symptoms are not unusual, affecting between 72 and 79% of GERD individuals (El-Serag et al., 2014, Shaker et al., 2003). GERD was found to be associated with different risk factors including socio-demographic variables, smoking, family history, high body mass index (BMI), types of food and drinks, fast food diets, physical activity and health conditions (Jarosz and Taraszewska, 2014, Saberi-Firoozi et al., 2007).

GERD may influence health-related quality of life and may induce psychological co-morbidities, impaired sleep quality, and absence from work with a relevant economic burden (Tack et al., 2012).

Different studies had found a significant correlation between age and GERD (He et al., 2010, Mohammed et al., 2003). Another study in KSA also showed that GERD was more prevalent in older persons (Almadi et al., 2014). However, other studies did not find any significant association between age and GERD prevalence (Altwigry et al., 2017, Chen et al., 2005, Nasseri-Moghaddam et al., 2008, Sharma et al., 2018). In few studies age was expressed as arbitrary cut-off (Chen et al., 2005, He et al., 2010, Nasseri-Moghaddam et al., 2008), whereas other studies expressed age as a continuous variable (Mohammed et al., 2003). This variation in the two methods might result in this difference between studies.

Food and diatry habits were linked to GERD in different studies. Spicy foods might induce heartburn as previously supported (Surdea-Blaga et al., 2017). Similarly, a study evaluated the role of daily consumed Korean traditional foods as well as westernized foods in GERD symptom generation (Choe et al., 2017). A study addressing soft drinks and GERD (Alkhathami et al., 2017) found significant association with a prevalence of GERD. This could be attributed to the highly acidic contents of soft drinks and the change in the intra-esophageal pH that might precipitate a GERD like symptoms, which may also increase acid-load in the stomach leading to increased probability of gastro esophageal reflux (Johnson et al., 2010). A study found a negative association between coffee and GERD symptoms (Murao et al., 2011). On the contrary, another study found a significant correlation between coffee drinking and GERD symptoms (Nandurkar et al., 2004).

The Aseer region is located in the Southwest of Saudi Arabia, bordering the northwestern region of Yemen. The area extends from high mountains chain called Sarawat down to the eastern coast of Red Sea. Abha is the capital of Aseer Province. It is famous for being the highest large city of Saudi Arabia at over 2200 m, some parts of the city being even around 2400 m. With such an altitude it is not surprising that Abha benefits from milder climatic conditions that most the rest of the country with day temperatures ranging from 15 °C to 35 °C. Data regarding prevalence and factors associated with GERD among attendants of primary health care centers of Abha city are scarce and even lacking. Therefore, this work was performed to study this problem.

## 2 Materials and methods

### 2.1 Study design

Abha city has 10 primary health care centers (PHCCs). A descriptive cross- sectional study targeted all adults attending Abha city PHCCs was performed.

### 2.2 Calculation of sample size

Using the WHO manual for sample size calculation in health sciences (Lwanga et al., 1991), with an estimate of 29% of GERD (Alsulobi et al., 2017), absolute required precision of 5% and 95% confidence interval, the calculated minimal sample size was 317 persons.

### 2.3 Sampling technique and data collection

The sample units were selected using stratified random sample technique. The sample was selected from the study 10 PHCCs taking into consideration the relative catchment population in each center (to avoid clustering effect). During the study period (late 2018 through early 2019) scheduled visits to the selected centers were arranged by the study field teams. During such visits, adult males and females attending the selected PHCCs for any reason (attending a clinic, accompanying their wife for her clinic, accompanying children for vaccination or well baby clinic visits, etc.) were invited to participate in the study and interviewed. A signed informed consent was obtained from each patient before inclusion in the study. Inclusion criteria (adults over 18 years) and exclusion criteria (pregnant women) were taken into consideration.

Data were collected using structured questionnaire. It comprised two parts. The first part included questions about the socio-demographic variables, life styles, dietary habits and health conditions of attendants (their records were also revised for past history). These included age, sex, work, education, smoking activity, fast food, type of food and chronic health disease. Details of diatry history and practicing exercise were assessed using a questionnaire interview. Body mass index (BMI) was estimated based on measured weight and height. Overweigh and obesity was considered when BMI was 25 kg/m^2^ or higher. The second part was the GerdQ that was a diagnostic for the GERD (Jones et al., 2009). The GerdQ consisted of six questions. Four questions were about the positive GERD predictors (heartburn, regurgitations of food or water, the disturbance in sleep due to the heartburn and regurgitation and taking medications). The other two questions were about the negative GERD predictors (nausea and epigastric pain). GerdQ scoring relies on the frequency of GERD symptoms during the last seven days (less than once, once, 2 times and 4–7 times, respectively). After summation of the Gerd scores, the patient who got score 8 or more was considered as having GERD (Jones et al., 2009). When the GerdQ was less than 8 it indicated low probability for GERD; while GerdQ ≥ 8 and ≤ 3 on questions 5 and 6 (sleep, heart burn and or regurgitation and medication) revealed GERD with low impact on daily life. Also, when GerdQ ≥ 8 and ≥ 3 on questions 5 and 6 indicated GERD with high impact on daily life.

### 2.4 Statistical analysis of data

Frequency and percent were used to describe different variables. Crude odds ratios (cOR) and concomitant 95% confidence intervals were calculated. Factors that were significantly associated with having GERD disease in bivariate analysis were included in a multiple logistic regression (Enter Model). Adjusted odds ratios (aOR) and concomitant 95% confidence intervals were computed. Selection of the variables to be included in multivariate regression model was based on purposeful selection of covariates (Bursac et al., 2008). The Hosmer–Lemeshow test was used to test goodness of fit for the logistic regression model.

## 3 Results

The present study included 320 persons attending PHCCs in Abha. The study showed that, overall, two out of each three persons were suffering from GERD (217, 67.8%). The prevalence of GERD with high impact on daily life (HIDL) was found to be 50% (160/320), while GERD with low impact on daily life (LIDL) was found to be 17.8% (57/320).

Table 1 shows the bivariate analysis of personal factors associated with GERD. Persons aged less than 30 years old were at a greater probability to have GERD compared to those aged 30 years and more (cOR = 2.06, 95% CI: 1.24–3.46). Similarly, the risk was greater among females, unmarried persons and those educated above secondary level. Students and those working in any job were significantly having more probability to have GERD compared to others.

**Table 1:** Personal data associated with GERD among study adult PHCCs attendants.

**Personal data**		**GERD (n = 320)**				**OR (95% CI)**
		**No GERD (n = 103)**		**GERD (n = 217)**		
		No	**%**	**No**	**%**	
**Age in years**	30+	67	39.4%	103	60.6%	Ref.**2.06 (1.24**–3.46) *
	Below 30	36	24.0%	114	76.0%	
**Gender**	male	62	37.1%	105	62.9%	Ref**1.6 (1.1**–2.6) *
	female	41	26.8%	112	73.2%	
**Marital Status**	Married	71	40.6%	104	59.4%	Ref2.41 (1.47-0.3.93) *
	Unmarried	32	22.1%	113	77.9%	
**Education level**	Below secondary	25	44.6%	31	55.4%	Ref**1.2 (1.1**–1.5) *
	Above secondary	78	29.5%	186	70.5%	
Work	Unemployed	43	45.3%	52	54.7%	Ref
	Student	56	28.1%	143	71.9%	2.1 (1.3–3.5)*
	Employed	4	15.4%	22	84.6%	4.5 (1.5–14.2)*
Body mass index	Normal (less than 25)	38	30.2%	88	69.8%	Ref0.86 (0.53–1.4)
	Overweight/ obese	65	33.5%	129	66.5%	

OR (95% CI) = Odds ratio (95% Confidence Interval), Ref = reference group, * Significant.

Table 2 shows the food items intake associated with GERD. Those who consumed fast food in the previous week had significantly higher probability to have GERD (cOR = 1.5, 95% CI:1.2–1.9). Similarly, spicy food and Arabic coffee were significantly associated with GERD.

**Table 2:** Food intake factors associated with GERD among study adult PHCCs attendants.

Food intake		GERD (n = 320)				OR (95% CI)
		**No GERD (n = 103)**		**GERD (n = 217)**		
		**No**	**%**	**No**	**%**	
Al-Kabsa	No	10	35.7%	18	64.3%	Ref1.1 (0.73–1.6)
	Yes	93	31.8%	199	68.2%	
Fried foods	No	25	39.1%	39	60.9%	Ref1.2 (0.91–1.6)
	Yes	78	30.5%	178	69.5%	
Fast foods	No	52	44.1%	66	55.9%	Ref**1.5 (1.2**–1.9) *
	Yes	51	25.2%	151	74.8%	
Spicy foods	No	45	38.5%	72	61.5%	Ref**1.3 (1.0**–1.6) *
	Yes	58	28.6%	145	71.4%	
Arabic coffee	No	15	42.9%	20	57.1%	Ref**1.3 (1.01**–1.8) *
	Yes	88	30.9%	197	69.1%	
fruits & vegetables	No	10	35.7%	18	64.3%	Ref1.0 (0.7–1.6)
	Yes	93	31.8%	199	68.2%	
Brown bread	No	33	28.9%	81	71.1%	Ref0.36 (0.69–1.2)
	Yes	70	34.0%	136	66.0%	
Soft drinks	No	52	37.1%	88	62.9%	Ref1.3 (0.96–1.5)
	Yes	51	28.3%	129	71.7%	
Grilled foods	No	15	34.9%	28	65.1%	Ref1.1 (0.76–1.5)
	Yes	88	31.8%	189	68.2%	

Al-Kabsa = traditional food composed of meat and rice, OR (95% CI) = Odds ratio (95% Confidence Interval), Ref = reference group, * Significant.

Table 3 shows life style conditions associated with GERD. The study showed that smokers have significantly more probability to have GERD compared to none-smokers (cOR = 1.3, 95% CI: 1.06–3.8). Walking and running for 30 min or more were found to have significantly less probability to have GERD compared to those who were not practicing (cOR = 0.67, 95% CI: 0.39–0.96).

**Table 3:** Lifestyle conditions associated with GERD among study adult PHCCs attendants.

Lifestyle		GERD (n = 320)				OR (95% CI)
		**No GERD (n = 103)**		**GERD (n = 217)**		
		**No**	**%**	**No**	**%**	
Smoking	Non-smoker	93	37.8%	190	62.2%	Ref**1.3 (1.06**–3.8) *
	Smoker	10	20.8%	27	79.2%	
walking/running	less than 30 min	70	29.8%	165	70.2%	**Ref0.67 (0.39**–0.96) *
	30 min or more	33	38.8%	52	61.2%	
swimming / exercises	less than 30 min	96	33.1%	194	66.9%	Ref1.6 (0.67–3.9)
	30 min or more	7	23.3%	23	76.7%	

OR (95% CI) = Odds ratio (95% Confidence Interval), Ref = reference group, * Significant.

Table 4 shows health conditions associated with GERD. Only subjective perception of stress was found to be significantly associated with GERD (cOR = 2.2, 95% CI: 1.3–3.6).

**Table 4:** History of chronic diseases associated with GERD among adult PHCCs attendants.

Chronic health problems		GERD (n = 320)				OR (95% CI)
		**No GERD (n = 103)**		**GERD (n = 217)**		
		**No**	**%**	**No**	**%**	
Bronchial Asthma	No	86	31.4%	188	68.6%	Ref0.78 (0.41–1.5)
	Yes	17	37.0%	29	63.0%	
Diabetes Mellitus	No	86	31.7%	185	68.3%	Ref0.87 (0.46–1.7)
	Yes	17	34.7%	32	65.3%	
High lipid profile	No	84	34.1%	162	65.9%	Ref1.5 (0.84–2.7)
	Yes	19	25.7%	55	74.3%	
Hypothyroidism	No	100	32.9%	204	67.1%	Ref2.1 (0.59–7.6)
	Yes	3	18.8%	13	81.3%	
High blood pressure	No	88	30.5%	200	69.5%	Ref0.47 (0.22 – 1.04)
	Yes	15	46.9%	17	53.1%	
Psychological disorders	No	85	31.4%	186	68.6%	Ref0.76 (0.40–1.4)
	Yes	18	37.5%	30	62.5%	
Subjective perception of stress	No	72	39.1%	112	60.9%	Ref**2.2 (1.3**–3.6) *
	Yes	31	22.8%	105	77.2%	

OR (95% CI) = Odds ratio (95% Confidence Interval), Ref = reference group, * Significant.

Table 5 shows multiple logistic regression model (Enter method) for predictors of GERD among PHCCs attendants. Only four significant independent factors associated with GERD were identified; being unmarried (aOR = 1.85, 95% CI:1.01–3.22); smoking (aOR = 2.11, 95% CI: 1.41–5.98), fast food intake (OR = 1.28, 95% CI:1.01–1.71) and subjective perception of stress (OR = 3.0. 95% CI: 1.68–5.26). Hosmer-Lemeshow chi-squared test (6.3) and a p-value (0.382) indicated that the model was a good fit with an accuracy of 73%.

**Table 5:** Multiple logistic regression for predictors of GERD among adult PHCCs attendants.

**Factors**	**B**	**S.E.**	**P**	**aOR**	**95% CI for aOR**	
					**Lower**	**Upper**
Age below 30 years	−0.83	0.34	0.086	0.43	0.28	1.64
Female gender	0.28	0.44	0.125	1.32	0.89	2.42
**Unmarried**	−0.61	0.30	0.047	**1.85**	**1.02**	**3.23**
Above secondary education	0.10	0.47	0.089	1.1	0.91	1.42
Work	0.58	0.34	0.088	1.78	0.92	3.41
**Fast food**	0.25	0.11	0.047	**1.28**	**1.01**	**1.71**
Spicy food	0.19	0.43	0.093	1.20	0.95	1.87
Arabic coffee	0.19	0.47	0.152	1.22	0.91	1.93
**Smoking**	0.74	0.36	0.006	**2.11**	**1.41**	**5.98**
Walking/ running 30 min/ more	−0.49	0.44	0.120	0.61	0.40	1.53
**Life stress**	1.10	0.28	0.001	**3.0**	**1.68**	**5.26**
Constant	−0.24	0.77	0.763	0.81		

B = regression co-efficient, SE = standard error, aOR = Adjusted Odds Ratio, 95% CI = 95% Confidence interval, Hosmer and Lemshow Test: χ^2^ = 6.3; P = 0.382; Model accuracy = 73.0%.

## 4 Discussion

The overall prevalence of GERD in the present study amounted to 67.8% (217 out of 320). The prevalence of GERD with high impact on daily life (HIDL) was found to be 50%. Nearly similar results (70.5%) were reported at Qassim region, Saudi Arabia (Altwigry et al., 2017). Lower prevalence of GERD was obtained in other regions of Saudi Arabia with a prevalence of 45.4% in Riyadh (Almadi et al., 2014) and 28.7% in Jizan (Alsuwat et al., 2018). A recent study in Jizan in 2019 using online questionnaire reported a prevalence of 32.2% (Kariri et al., 2020). Similarly, a recent cross-sectional study among King Khalid University students in 2019 (Awadalla, 2019) reported a prevalence rate of GERD of 33.2%. Another cross-sectional study among medical students of King Abul-Aziz University of Jeddah in 2019 identified a GERD prevalence of 25.9% (Atta et al., 2019). The variations of the prevalence of GERD different regions of Saudi Arabia can be attributed to geographical differences in the prevalence of GERD risk factors (e.g., smoking, obesity, chronic diseases, etc), demographic (gender and age distribution) and life style conditions (dietary habits and physical inactivity).

On the other hand, low prevalence ranges of GERD were detected in North America (18.1–27.8%); Europe (8.8–25.9%); East Asia (2.5–7.8%); Middle East (8.7–33.1%); Australia (11.6%) and South America (23%) as previously reported (El-Serag et al., 2014). These variations might be attributed to differences in the investigated demographic factors, life styles, dietary habits and health conditions.

In bivariate analysis, the present study identified the following associated factors with GERD; age, gender, marital status, level of education, work status, smoking, intake of fast food, intake of spicy food, intake of Arabic Coffee, smoking and, lack of excercise (walking), and subjective perception of stress.

On the other hand, in multiple logistic regression analysis only four associated risk factors with GERD were found. They were being unmarried, intake of fast foods, smoking and subjective perception of stress. A recent study in the Western region of Saudi Arabia among Taif University students (Elnemr et al., 2018) reported similar findings, where the predictors of GERD among university students were stress, intake of spicy food and smoking.

The marital status was a significant factor associated with frequency of GERD. A higher prevalence of high impact GERD was found in single, followed by divorced/ widow and then in married ones. This relationship between GERD and marital status has been reported by Jizan study (Kariri et al., 2020). Similarly, most of previous studies found a significant correlation between GERD and marital status (Pourhoseingholi et al., 2012, Shaha et al., 2012). In Saudi Arabia, a higher significant prevalence of GERD was reported among divorced/ widow followed by singles (Alkhathami et al., 2017). We could summarize an explanation to the higher prevalence of GERD among singles and divorced/ widows to the psychological stresses which could develop symptoms of GERD (Naliboff et al., 2004).

Concerning dietary habits, fast food was a significant factor associated with a higher prevalence of GERD among individuals consuming fast food in our study. Similar results in Al Taif (Alkhathami et al., 2017) was found where a significant relationship between the type of food, GERD symptoms and increased risk of reflux in those who consume fast food in a regular pattern. Similarly in Jizan, GERD was significantly associated with those who regularly consumed fast food. (Kariri et al., 2020) This result was previously reported in studies performed in Turkey (Chirila et al., 2016) and in Iran (Khodarahmi et al., 2016), where the fast food consumption was a significant risk factor for acid reflux.

Regarding life style, and similar to previous studies that reported that smoking had been identified as a risk factor of GERD (Alkhathami et al., 2017, Nouraie et al., 2007), where high prevalence of GERD was found in smokers compared to lower rates in non-smokers. Similar findings were reported in Jizan populations (Kariri et al., 2020), King Khalid University students (Awadalla, 2019) and Jeddah Medical students (Atta et al., 2019). Moreover, the benefits of smoking cessation in control of GERD symptoms have been also reported by previous studies (Kohata et al., 2016). Smoking affects the GERD through impairing lower esophageal sphincter action which is an important barrier to acid reflux by decreasing its pressure (Smit et al., 2001).

In the current study, the perceived stress was a significant factor associated with the prevalence of GERD, where the participants having life stresses showed a higher frequency of GERD compared to a lower frequency in those without any stress. Similar results were found among University students (Awadalla, 2019). Similarly, finding in Korea revealed that GERD was a significantly associated with psychosocial stress, and severity of reflux esophagitis correlates with the degree of stress (Song et al., 2013). It has been noticed that high levels of stress hormones might slow down the gastric emptying (Gue et al., 1989). This could allow for an increase in the gastric acid and gas production, therefore, possibly propelling the stomach contents into the esophagus, resulting in GERD development.

This was the first work in Abha city, southwestern Saudi Arabia to investigate the prevalence of GERD and to correlate it with some important factors. However, the study has some limitations. As a cross-sectional design, the direction of the association between studied factors and GERD cannot be properly established. Also, the study was performed at PHCC level may not fully represent the gen eral poulation. Another restriction of the study was related to the fact that the data were obtained by interview questionnaire. Answers may have been distorted by recall bias.

## 5 Conclusions

The overall prevalence of GERD in the present study amounted to 67.8%. This study confirmed a higher prevalence of high impact GERD compared to a lower frequency of low impact GERD among PHCCs attendants at Abha city, KSA based on GerdQ score. This study illustrated that unmarried, fast food, spicy food, smoking, life stress were positive predictors of GERD among attendants of PHCCs at Abha city, KSA. GERD is a public health problem among adults in the region. Community level awareness programs are recommended. Healthcare providers must be aware of community perceptions and practices.

## 6 Authors’ contributions

All contributing authors have agreed to the submission of this manuscript for publication. SAA, NJA, AAM, MAA designed and were major contributors in writing the manuscript. MAA, MMA, MSA, FSA, MSA, ASA acquired data. SFS performed the statistical analysis of data. All authors have read and approved the final version of this manuscript.

## Funding

The study was self-funded.

## Declaration of Competing Interest

The authors declare that they have no known competing financial interests or personal relationships that could have appeared to influence the work reported in this paper.
